# Classification and characterization of electromagnetic materials

**DOI:** 10.1038/s41598-020-68298-3

**Published:** 2020-07-09

**Authors:** Yosef T. Aladadi, Majeed A. S. Alkanhal

**Affiliations:** Department of Electrical Engineering, King Saud University, Riyadh, 11421 Saudi Arabia

**Keywords:** Engineering, Electrical and electronic engineering, Materials science

## Abstract

In this paper, we present an efficient method to classify complex electromagnetic materials. This method is based on the directional interaction of incident circularly polarized waves with the materials being tested. The presented method relies on an algorithm that classifies the test materials to one of the following categories: isotropic, chiral, bi-isotropic, symmetric anisotropic or general bianisotropic. The transmitted and reflected fields of right-handed and left-handed circularly polarized waves normally incident from three orthogonal orientations are utilized to determine the reflection/transmission coefficients and complex refractive indices. Both analytical and numerical solutions are used to compute fields of the circularly polarized waves from the arbitrary complex material slab. The complex materials are discriminated accordingly and then classified under an appropriate category. Additionally, new results for material characterization by extracting the scalar/tensorial parameters of bi-isotropic and gyrotropic materials are presented.

## Introduction

Recent advances in the interaction of electromagnetic fields with complex composite media suggest the feasibility of creating novel materials with unusual electromagnetic properties and the possibility of constructing new electromagnetic devices using such materials. These improved materials can be engineered to possess several unique electromagnetic properties that make them suitable candidates for numerous applications in modern technology systems. Applications related to the fields of photonics^1^, optoelectronics^2,3^, radar cross-section reduction^4–7^, gigahertz devices^8^, antenna reconfiguration^9^, terahertz plasmonic filters^10^, and biosensors^11^ are the leading beneficiaries of such developments. These complex electromagnetic materials are often further classified to isotropic, bi-isotropic, anisotropic and bianisotropic based on their macroscopic electromagnetic properties^12–14^, which provide the description of certain materials through their constitutive relations.

Isotropic materials like unstressed glass and plastic, water and air, and fluids at rest, behave precisely in the same manner regardless of the direction of the wave propagation axis, because their permittivities and permeabilities are identical in all directions. In contrast, in anisotropic materials like wood, the electromagnetic properties are different in different directions. Materials with crystalline structure are biaxial anisotropic materials. For example, yttrium orthosilicate (YSO) is a dielectric material with biaxial anisotropy at optical frequencies. This well-known rare-earth host material has shown promising performance in quantum-engineered optical devices development^15^. Yttrium aluminum perovskite (YAP) crystal material, with orthorhombic symmetry, is designated as a symmetric anisotropic material^16^. Gyrotropic magnetized ferrites are asymmetric anisotropic materials that have been used in microwave engineering for years because of their non-reciprocal behavior that makes them very useful in the design of microwave devices like isolators, polarizers, and circulators^17^. Graphene in the static magnetic field is a gyrotropic and uniaxial anisotropic material in the absence of the external magnetic field^18^. More recently, materials with optical activity (chirality) have been considered for application in microwave and infrared regions. Chiral materials are a particular case of the more general bi-isotropic materials. A chiral composite can be constructed by embedding chiral objects such as wire helixes, Möbius strips, or irregular tetrahedrons in a nonchiral host isotropic medium^19^. A medium is bianisotropic when its constitutive relations are generalized 3 × 3 matrices or tensors. An artificial composite metamaterial composed of split ring resonators with rods^20^ can be modeled as bianisotropic material. Efficient methods for the classification and characterization of such materials based on determining their macroscopic electromagnetic properties are of growing importance but still in evolution^12–14^. The traditional characterization techniques used to measure the electromagnetic properties of scalar and tensorial permittivity and permeability of complex materials have received significant efforts in recent years^1,3,21–31^. These techniques are, essentially, based on measuring the scattering parameters from the investigated materials in free space^22,28^ using single or multiple normal^32^ and oblique^33^ linearly polarized wave incidence. The characterization procedures start from deducing the effective refractive index and effective wave impedance from the transmitted and reflected fields. However, these methods are liable to obtaining multiple branching ambiguity, which limits using them at high frequencies, especially for thick material slabs. In addition, the methods that use optimization schemes are usually accompanied by heavy computation costs and often yield multiple solutions. Efforts have been introduced to overcome these discontinuities based on the Kramers–Kronig (K–K) relation^22,34^, and phase correction techniques^35–37^. However, the K–K method is saturated at high frequencies, which limits its performance especially for thick structures. The phase correction techniques are sensitive to simple errors in phase data and they, also, are susceptible to slip to error solutions at zero refractive index values or if they were initialized at an arbitrary starting frequency^36^. The existing characterization methods attempt to find the practicable effective (equivalent) scalar and/or tensorial parameters that model the test material regardless of their essential classification or discrimination according to their inherent electromagnetic directional behavior.

This paper presents a novel method to classify complex electromagnetic materials based on their behavioral (directional) interaction with incident circularly polarized waves. The method is based on a classification algorithm of the unknown materials to one of the following categories: isotropic, chiral, bi-isotropic, symmetric anisotropic, or general bianisotropic. This method utilizes the scattering parameters of simple normal-incident (LCP/RCP) circularly polarized plane wave measurements from three different orthogonal axes and their corresponding refractive indices. The proposed scheme is a simple and direct classification process without demanding a complete intricate extraction process for the tensor elements that are usually accompanied by multiple solutions that need carful processing. Beside that, after classification, the characterization process (extraction of the tensor elements) becomes much easier when the investigated material model is predetarmined. Solutions from an analytical method based on the transmission matrix method (TMM) and numerical results from a full wave simulator are used to compute the transmitted and reflected fields of the circularly polarized waves from the arbitrary complex material slab. The different complex materials are discriminated accordingly and then classified under an appropriate category. Additionally, new accurate material characterization results are obtained by retrieving the scalar/tensorial parameters for bi-isotropic and asymmetric anisotropic materials.

## Classification algorithm

Using Fresnel–Airy formulas, the complex wave impedance ($Z=R+jX$) and complex refractive index ($n={n}^{\prime}+j{n}^{\prime\prime}$) are described in terms of the reflection and transmission coefficients for circularly polarized waves as follows:

1 $${Z}^{_{-}^{+} }=\pm \sqrt{\frac{{\left(1+{{S}_{11}}^{_{-}^{+} }\right)}^{2}-{{{S}_{21}}^{_{-}^{+} }}^{2}}{{\left(1-{{S}_{11}}^{_{-}^{+} }\right)}^{2}-{{{S}_{21}}^{_{-}^{+} }}^{2}}}$$

2 $${n}^{_{-}^{+} }=\frac{-j}{{k}_{0}d} ln\left(\frac{{{S}_{11}}^{_{-}^{+} }}{1-{{S}_{21}}^{_{-}^{+} }\left(\frac{{Z}^{_{-}^{+} }-1}{{Z}^{_{-}^{+} }+1}\right)}\right),$$

where $"+"$ and $"-"$ denote the right circularly polarized (RCP) and left circularly polarized (LCP) waves respectively. The sign of $Z$ is determined according to the passive material conditions ( $R\ge 0$, ${n}^{\prime\prime}\ge 0$)^32^. ${{S}_{11}}^{_{-}^{+}}$ and ${{S}_{21}}^{_{-}^{+}}$ are the circular polarization reflection and transmission coefficients.

**Figure 1:**
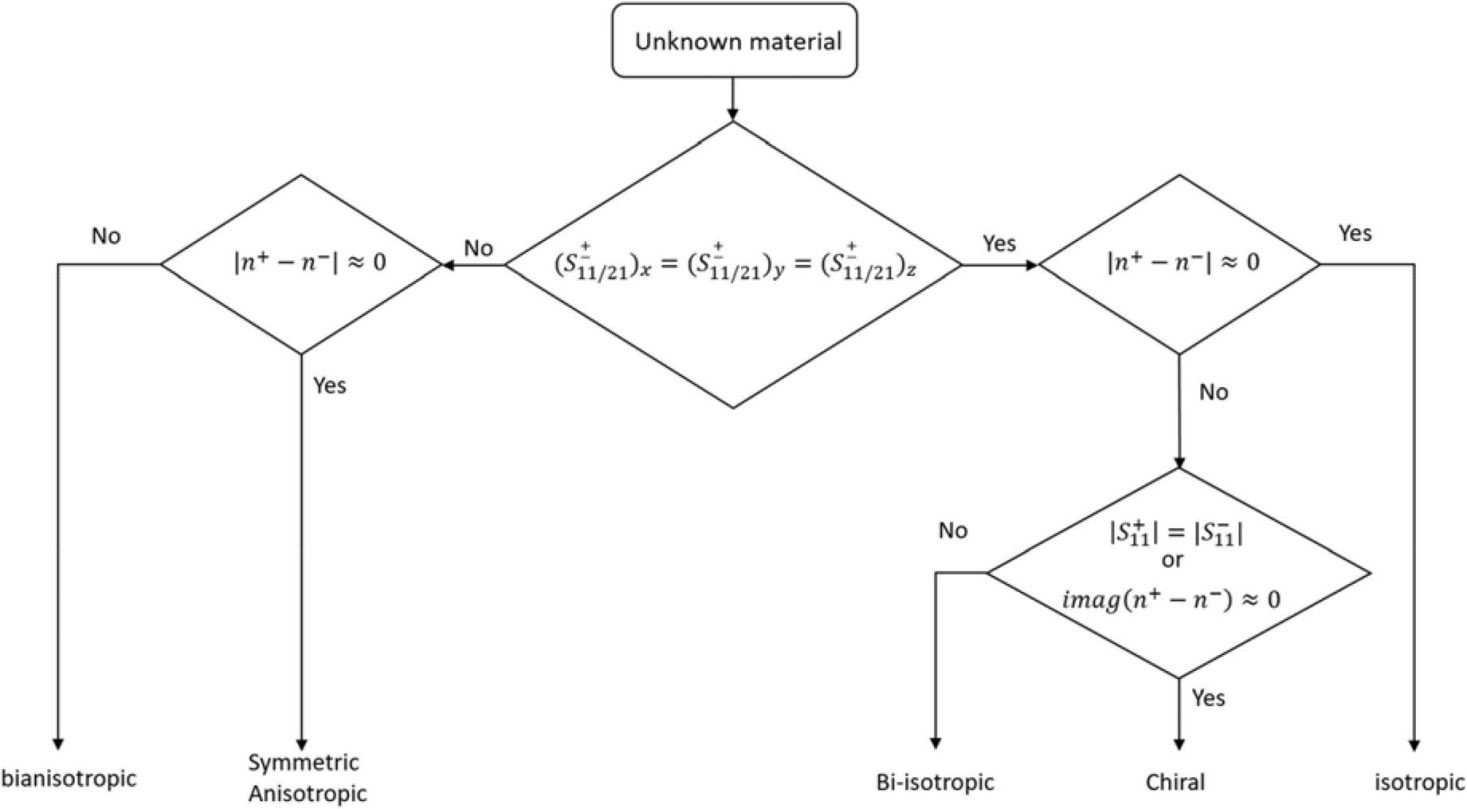
Material classification algorithm.

Both analytical and numerical solutions are used to obtain the RCP and LCP transmitted and reflected electromagnetic fields from the investigated materials. The analytical solutions use the TMM^38^, and the numerical solution uses a full wave simulator. Electromagnetic fields penetrating the bianisotropic/anisotropic materials display a number of characteristic and diagnostic effects, which are subtly different from those of fields propagating in the bi-isotropic, chiral or isotropic materials. Bianisotropic/anisotropic materials are those whose properties are directionally dependent, unlike bi-isotropic/isotropic materials that have their material properties identical in all directions. This means that bianisotropic/anisotropic materials are sensitive to change in the propagation axis whereas the bi-isotropic\isotropic materials are not. Figure 1 shows the proposed classification algorithm. It is based on normal RCP or LCP field measurements in three orthogonal propagation axes. If the reflection or transmission coefficients of the incident RCP or LCP waves in these axes are the same, then the investigated material belongs to the general bi-isotropic structure. If not, the sample is a general bianisotropic material. For general bi-isotropic structures, if the refractive indices related to the RCP and LCP waves are similar, then the material is simply isotropic. If not, the material is bi-isotropic/chiral. If the imaginary part of the refractive indices related to the RCP and LCP are the same, then the material is chiral^39^. The second phase of classification is based on discrimination between symmetric anisotropic and bianisotropic/asymmetric anisotropic materials. If $\left|{n}^{+}-{n}^{-}\right|\approx 0$ for all the three propagation axes, then the investigated material is symmetric anisotropic as in biaxial materials^40,41^. If $\left|{n}^{+}-{n}^{-}\right|\ne 0$ at least for one propagation axis, the material is classified as asymmetric or bianisotropic.

## Electromagnetic fields in complex materials

In this section, the scattering parameters of the selected materials are determined using analytical or numerical solutions. Bi-isotropic media are the most general linear, homogenous and isotropic materials. The constitutive relations that define the bi-isotropic media are given by

3 $$ \begin{aligned} & D = \varepsilon E + \xi H \\ & B = \zeta E + \mu H, \\ \end{aligned} $$

with $\varepsilon$ and $\mu$ being the scalar permittivity and permeability of the material, respectively, and $\zeta$ and $\xi$ are the magnetoelectric coupling coefficients. Based on the law of energy conservation^41^, it is required that the bi-isotropic coefficients be generally expressed as $\zeta={\xi }^{*}= \chi + j\kappa$, where $\chi$ is the Tellegen coefficient, and $\kappa$ is the chirality coefficient.

Biaxial and gyrotropic media are used in this study as illustrations for symmetric and asymmetric anisotropic materials, respectively. Gyrotropic media is defined by permittivity and permeability tensors as

4 $$\varepsilon =\left[\begin{array}{ccc}{\varepsilon}_{1}& j{\varepsilon }_{2}& 0\\ -j{\varepsilon }_{2}& {\varepsilon }_{1}&0\\ 0& 0& {\varepsilon}_{3}\end{array}\right],$$

5 $$\mu =\left[\begin{array}{ccc}{\mu }_{1}& j{\mu }_{2}& 0\\ -j{\mu }_{2}& {\mu }_{1}& 0\\ 0& 0& {\mu }_{3}\end{array}\right].$$

For a lossless medium, the permittivity and permeability matrices are Hermitian and positive-definite^41^, which implies that ${\varepsilon }_{1},{\varepsilon }_{2}$, ${\varepsilon }_{3}$ $, {\mu }_{1},{\mu }_{2}$ , ${\mu }_{3}$ are real, and moreover ${\varepsilon }_{1}$>0, |${\varepsilon }_{2}$|≤ 1, ${\mu }_{1}$>0, |${\mu }_{2}$|≤ 1, ${\varepsilon }_{3}$>0 and ${\mu }_{3}$>0.

The permittivity and permeability tensors of the biaxial media are:

6 $$\varepsilon =\left[\begin{array}{ccc}{\varepsilon }_{x}& 0& 0\\ 0& {\varepsilon }_{y}& 0\\ 0& 0& {\varepsilon }_{z}\end{array}\right],$$

7 $$\mu =\left[\begin{array}{ccc}{\mu }_{x}& 0& 0\\ 0& {\mu }_{y}& 0\\ 0& 0& {\mu }_{z}\end{array}\right].$$

**Figure 2:**
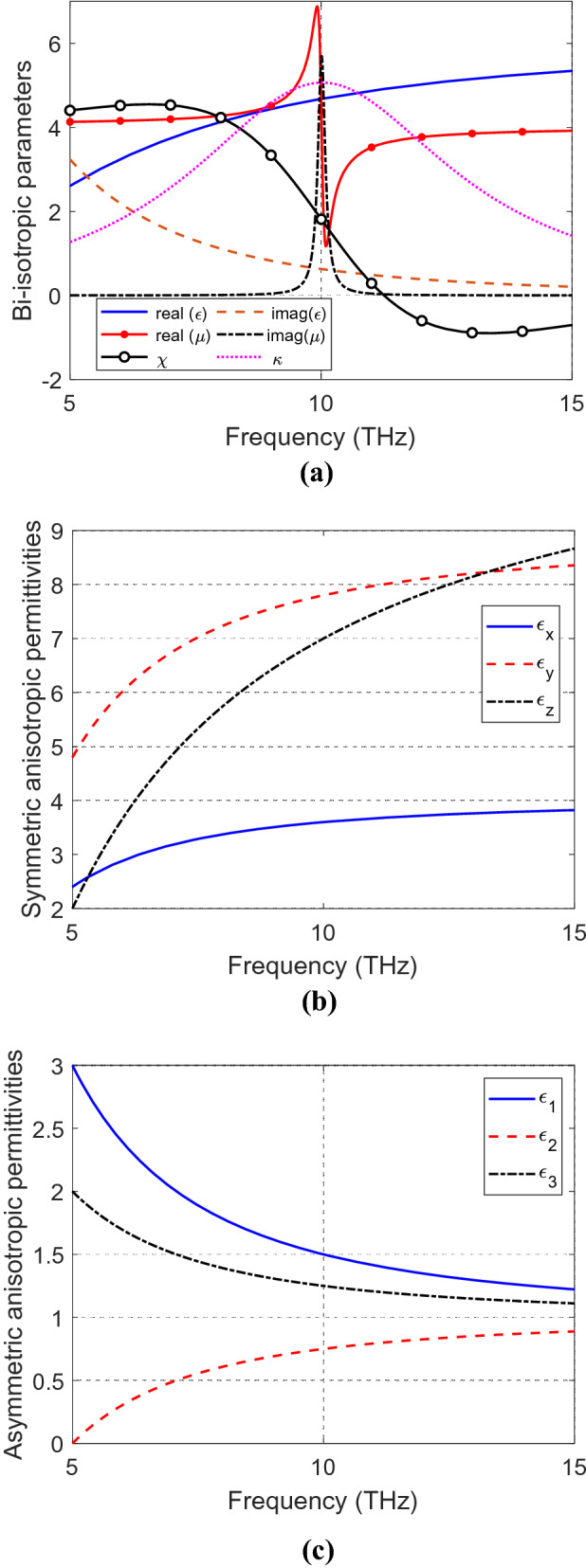
Parameters of illustrated (**a**) bi-isotropic and chiral materials, (**b**) symmetric anisotropic material, and (**c**) asymmetric anisotropic material.

Recently, researches focus their efforts on the study of the interaction of electromagnetic fields with complex materials in the terahertz frequency ranges for possible new applications and devices. Terahertz investigations of structures like graphene-dielectric stacks^42^, metal and graphene hybrid metasurface^43^, organic crystals for THz photonics^44^, terahertz metamaterials^45,46^ and chiral metamaterials^46^ are few examples of such efforts. The methods presented in this paper are general and can be applied to any frequency band; still it is demonstrated here at the terahertz bands. Drude and Lorentz Models^22^ are used to select parameters of the tested bi-isotropic material as shown in Fig. 2a. The permittivity is modelled using the Drude module, whereas the permeability and the magnetoelectric coupling coefficients are modelled using the Lorentz model. For chiral material, the Tellegen coefficient is set to zero, and the other parameters are similar to those of the Tellegen (general bi-isotropic) material. The selected permittivities of the symmetric and asymmetric anisotropic materials are based on Drude^22^ and Debye^47^ models, respectively, as shown in Fig. 2b, c. The permeabilities of the symmetric and asymmetric anisotropic material are considered as unity in this investigation.

Three different orthogonal measurements of the RCP and LCP waves transmitted, and reflected electromagnetic fields from the investigated materials are engaged. Theoretically, the orientations of the slab axes are described by the Euler’s angles^48^, $\theta$, $\phi$, $\psi$, with respect to the fixed *xyz* coordinates. The dielectric tensor after rotation in the *xyz* coordinate system is given by

8 $$\stackrel{\sim }{\varepsilon }=R\varepsilon {R}^{-1}$$

9 $$\stackrel{\sim }{\mu }=R\mu {R}^{-1},$$

where R is the coordinate rotation matrix, given by

10 $$ R = \left( {\begin{array}{*{20}c} {cos\varPsi cos\phi - cos\theta sin\phi sin\psi } & { - sin\psi cos\phi - cos\theta sin\phi cos\psi } & {sin\theta sin\phi } \\ {cos\varPsi sin\phi + cos\theta cos\phi sin\psi } & { - sin\psi sin\phi + cos\theta cos\phi sin\psi } & { - sin\theta cos\phi } \\ {sin\theta sin\psi } & {sin\theta cos\psi } & {cos\theta } \\ \end{array} } \right) $$

**Figure 3:**
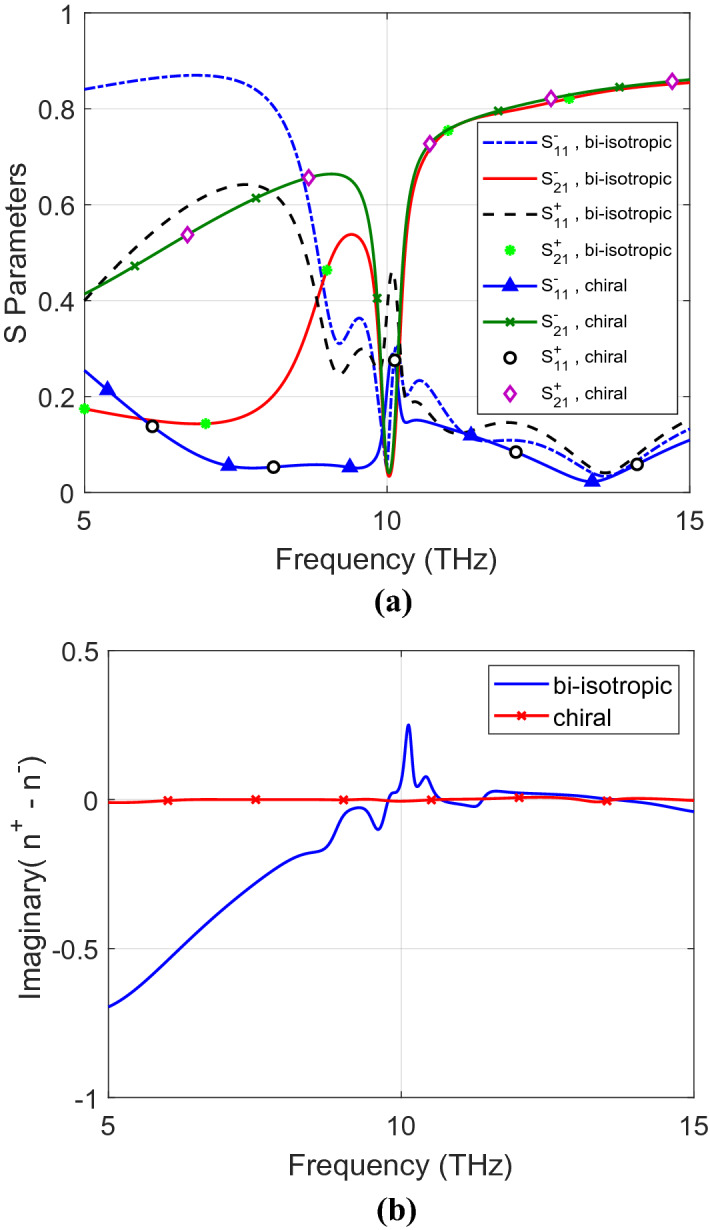
(**a**) Reflection and transmission coefficients and (**b**) imaginary part of refractive index of chiral and bi-isotropic materials for $x,y$ and $z$ propagation axes for both RCP and LCP polarizations.

The corresponding transmission and reflection coefficients of the 5 µm thick chiral and bi-isotropic materials are determined analytically as shown in Fig. 3a. The results are demonstrated for the three *xyz* propagation axes for both LCP and RCP waves. The scattering parameters of both chiral and bi-isotropic materials are the same for different propagation axes. However, the bi-isotropic material is sensitive to the polarization that appears obviously in different LCP and RCP S-parameters. In addition, the difference of the imaginary parts of the refractive indices of the RCP/LCP measurements differentiate between chiral and bi-isotropic materials as shown in Fig. 3b. It is zero for chiral materials and nonzero for bi-isotropic materials as described in the classification algorithms. If the slab is very thin, differences (contrast) in the values of the RCP and LCP S-parameters is likely to be small.

**Figure 4:**
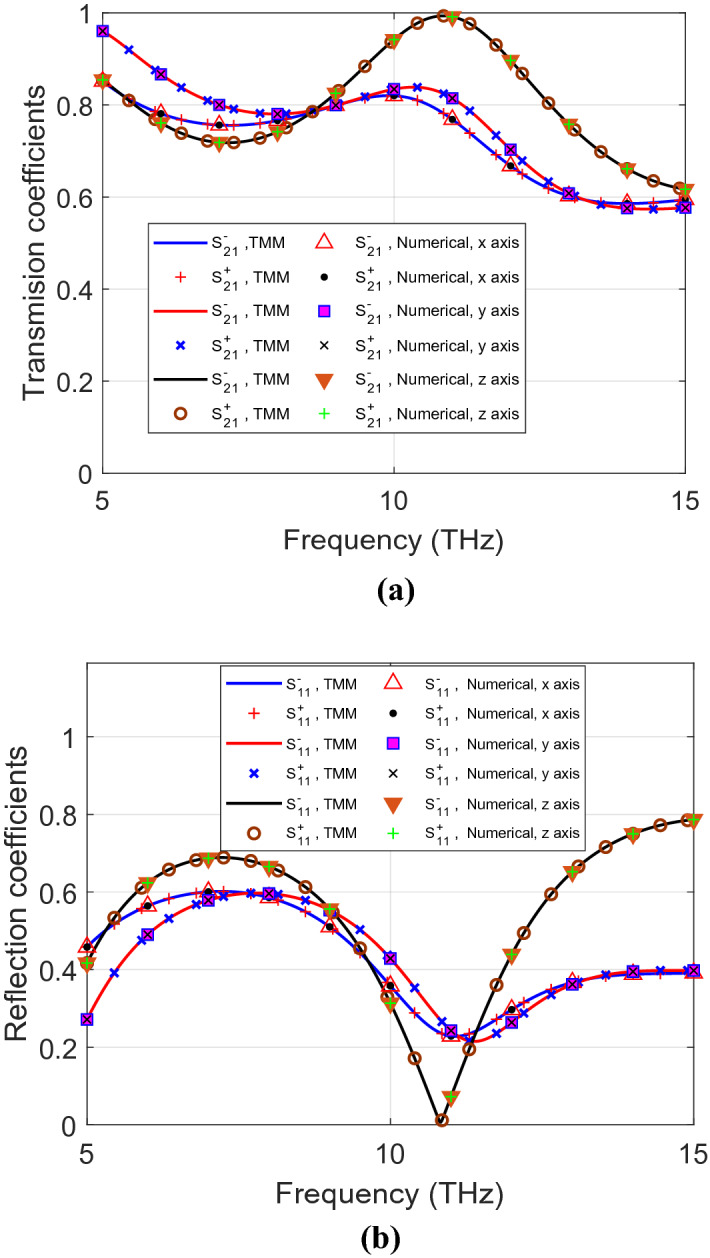
(**a**) Transmission and (**b**) reflection coefficients of symmetric anisotropic materials for LCP and RCP polarization for *xyz* propagation axes, using both analytical and numerical methods.

**Figure 5:**
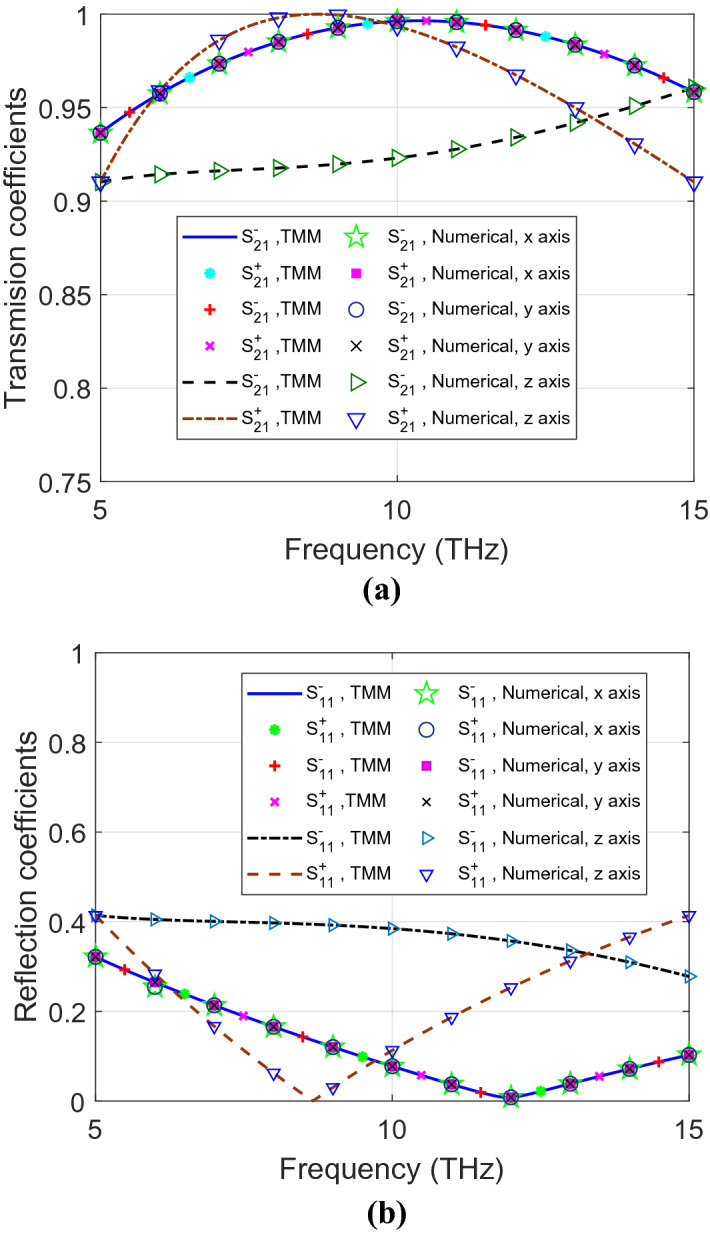
(**a**) Transmission and (**b**) reflection coefficients of asymmetric anisotropic materials for LCP and RCP polarization for *xyz* propagation axes, using both analytical and numerical methods.

**Figure 6:**
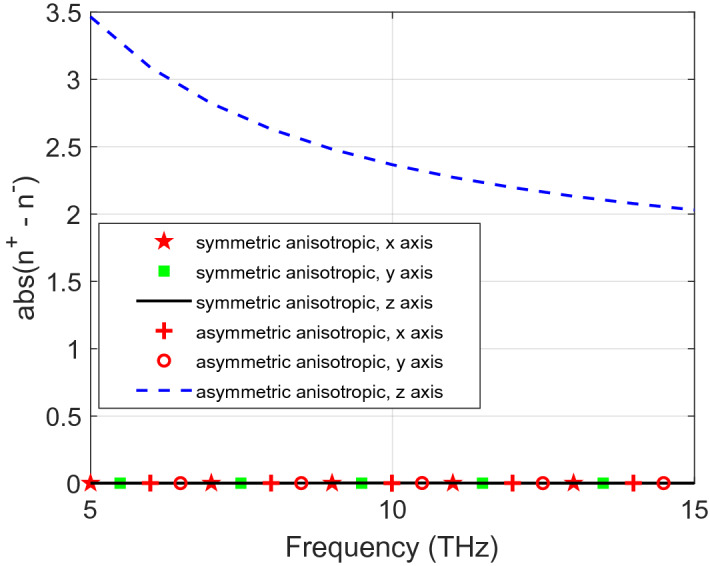
Absolute difference of refractive indices of RCP and LCP of symmetric and asymmetric anisotropic materials for three measurements of x, y and z propagation axes.

Figures 4 and 5 show RCP and LCP transmission and reflection coefficients for symmetric and asymmetric materials, respectively, from the analytical and the numerical solutions. Both of them are dependent on the orientation of the propagation axis. For the symmetric anisotropic case, ${S}_{11}^{+}$ is the same as ${S}_{11}^{-}$ for all the three propagation axes. However, for asymmetric materials, ${S}_{11}^{+}$ and ${S}_{11}^{-}$ are not the same for the *z* propagation axis. In addition, $\left|{n}^{+}-{n}^{-}\right|\ne 0$ in the *z* propagation axis indicates asymmetry of this material as shown in Fig. 6. This information can help to classify the investigated material as an asymmetric anisotropic material.

## Bi-isotropic and gyrotropic material characterization

After the classification of complex materials, characterization of electromagnetic materials becomes easier. In this section, we present new results for the extraction of the scalar/tensorial parameters of bi-isotropic and gyrotropic materials. For bi-isotropic materials, the permittivity and permeability are defined as^49^

11 $$\varepsilon =\frac{{\varepsilon }^{+}+{\varepsilon }^{-}}{2},$$

12 $$\mu =\frac{{\mu }^{+}+{\mu }^{-}}{2}.$$

In this research, the Tellegen and chirality coefficients are expressed more accurately as

13 $$\chi + j\kappa =j\frac{{ z}^{2}\left({ z}^{+}{ n}^{+}-{ z}^{-}{ n}^{-}\right)+{ z}^{+}{ n}^{-}-{ z}^{-}{ n}^{+}}{{ 4z}^{2}}$$

Here, $${\varepsilon }^{\pm }=\frac{{n}^{\pm }}{{z}^{\pm }}$$ and $${\mu }^{\pm }={n}^{\pm } {z}^{\pm }$$.

For the *z* propagation axis, the parameters of the gyrotropic material are given as^41,49^

14 $${\varepsilon }_{1}=\frac{{\varepsilon }^{+}+{\varepsilon }^{-}}{2},$$

15 $${\varepsilon }_{2}=\frac{{\varepsilon }^{+}-{\varepsilon }^{-}}{2}$$

For the *x* or *y* propagation axes, the third permittivity element can be determined as

16 $${\varepsilon }_{3}={\varepsilon }^{+}+{\varepsilon }^{-}-{\varepsilon }_{1}$$

**Figure 7:**
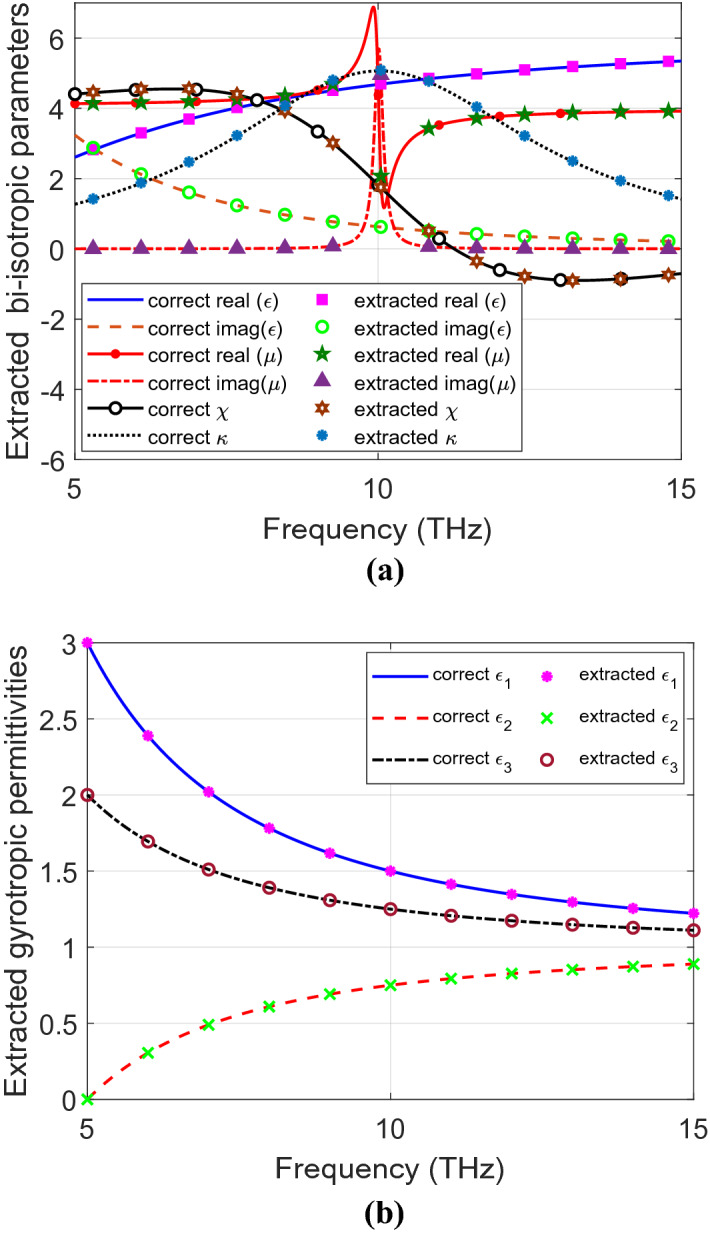
Extracted parameters of the tested (**a**) bi-isotropic material (**b**) gyrotropic material.

Figure 7a shows the extracted parameters of the illustrated bi-isotropic material from the transmitted and the reflected fields. Figure 7b depicts the extracted parameters of the tested gyrotropic material. The extracted parameters agree with the correct parameters in the numerical scales of the presented figures.

## Conclusions

This paper presented an efficient method to classify complex electromagnetic materials based on their directional interaction with incident circularly polarized waves. The method used an algorithm that classifies unknown materials to one of the following categories: isotropic, chiral, bi-isotropic, symmetric anisotropic or general bianisotropic. The required reflection/transmission coefficients and complex refractive indices were inferred from the transmitted and reflected fields of LCP/RCP circularly polarized waves normally incident from three different orientations. Solutions from an analytical method based on the transmission matrix method (TMM) and numerical results from a full wave simulator were used to compute the fields of the circularly polarized waves from the arbitrary complex material slab. The complex materials were discriminated accordingly and classified under an appropriate category. Furthermore, new accurate material characterization results were acquired by retrieving the scalar/tensorial parameters of bi-isotropic and asymmetric anisotropic (gyrotropic) materials.
